# MicroRNA-223 restricts liver fibrosis by inhibiting the TAZ-IHH-GLI2 and PDGF signaling pathways via the crosstalk of multiple liver cell types

**DOI:** 10.7150/ijbs.58365

**Published:** 2021-03-11

**Authors:** Xiaolin Wang, Wonhyo Seo, Seol Hee Park, Yaojie Fu, Seonghwan Hwang, Robim M. Rodrigues, Dechun Feng, Bin Gao, Yong He

**Affiliations:** 1 Laboratory of Liver Diseases, National Institute on Alcohol Abuse and Alcoholism, National Institutes of Health, Bethesda, MD 20892, USA.; 2 Department of Infectious Diseases, Ruijin Hospital, Shanghai Jiao Tong University School of Medicine, Shanghai, 200025, China.

**Keywords:** Liver fibrosis, HSC, miR-223, Gli2, PDGFRα/β

## Abstract

**Background & Aims:** Liver fibrosis is a common consequence of chronic liver injury and is characterized by the accumulation of extracellular matrix mainly generated from activated hepatic stellate cells (HSCs). At present, the mechanisms underlying liver fibrogenesis remain obscure and effective pharmacological therapies are lacking. Neutrophil-specific microRNA-223 (miR-223) plays an important role in controlling the development of various liver diseases; however, its role in HSC activation and liver fibrosis remains unclear.

**Methods:** Liver fibrosis was induced by chronic carbon tetrachloride (CCl_4_) injection of miR-223 knockout (miR-223KO) mice and littermate wild-type controls. MiR-223 was overexpressed in cultured HSCs to determine its function and targets during HSC activation and proliferation. The expression of miR-223 and pri-miR-223 was examined in primary HSCs isolated from CCl_4_-treated mice and in cultured HSCs. The communication between HSCs and neutrophils was studied by performing *in vitro* co-culture experiments.

**Results:** Genetic deletion of miR-223 exacerbated chronic CCl_4_-induced liver fibrosis. Administration of miR-223 inhibited liver fibrosis by inhibiting the transcriptional coactivator with PDZ-binding motif (TAZ)-Indian hedgehog (IHH)-GLI Family Zinc Finger 2 (GLI2) pathway via the crosstalk between hepatocytes and HSCs. Overexpression of miR-223 also directly attenuated *Gli2* as well as platelet-derived growth factor receptor α/β (*Pdgfra/b*) expression in HSCs, thereby suppressing HSC activation and proliferation. The expression of pri-miR-223 and miR-223 was downregulated during HSC activation *in vitro*. Expression of pri-miR-223 was also decreased in activated HSCs *in vivo* in fibrotic livers but mature miR-223 expression was not reduced. Finally, in co-culture experiments, activated HSCs were able to take up miR-223-enriched extracellular vesicles from neutrophils, resulting in elevation of miR-223.

**Conclusion:** MiR-223 restricts liver fibrosis by targeting multiple genes in hepatocytes and HSCs, providing potential therapeutic targets for the treatment of liver fibrosis.

## Introduction

Hepatic fibrosis is a dynamic process characterized by the net accumulation of extracellular matrix (ECM) and myofibroblast-rich scar tissue, caused by chronic liver injury, including chronic viral infection, alcoholic liver disease, nonalcoholic steatohepatitis (NASH), and autoimmune hepatitis 1. Of note, liver fibrosis can progress to liver cirrhosis and liver cancer but its mechanisms remain obscure and effective pharmacological therapies are lacking 2.

Activation of hepatic stellated cells (HSCs) is the key step in the development of liver fibrosis 3. Following liver injury or culture *in vitro*, HSCs transdifferentiate from a quiescent phenotype into ECM-producing myofibroblasts, serving as the major source of ECM production in liver fibrosis 4. A variety of fibrogenic mediators and their downstream signaling pathways have been identified to drive HSC activation, such as transforming growth factor β (TGFβ), platelet-derived growth factor (PDGF), connective tissue growth factor, and the Hedgehog pathway 1. Immune cell-mediated liver inflammation is well known to play an important role in regulating HSC activation. For example, macrophages contribute to liver fibrogenesis by secreting TGFβ, which is the most potent fibrogenic cytokine for HSC activation 5. Neutrophils likely promote liver fibrosis via production of ROS but may also promote liver fibrosis resolution by transferring microRNA into macrophages 6-9. Moreover, natural killer cells have been reported to kill activated HSCs, thereby inhibiting liver fibrosis 10. Up till now, there are no clinically effective ways to inhibit HSC activation and reduce liver fibrosis. Therefore, understanding of the regulatory mechanisms involved in HSC activation could provide new insights for the treatment of liver fibrosis.

MicroRNAs (miRNAs), which are endogenous, noncoding, single-stranded RNAs of ~22 nucleotides in length, play a key role in controlling multiple physiological processes by negatively regulating target gene expression through complementary binding with the 3' untranslated regions (UTRs) 11-13. Accumulating studies have demonstrated the important roles of miRNAs in the progression of liver diseases 13,14. Moreover, miRNAs can be packaged and released in extracellular vehicles (EVs), serving as a messenger for cell-to-cell communication 13. MiR223 is a myeloid cell-specific miRNA which is expressed at the highest level in neutrophils and is specifically enriched in neutrophil/myeloid cell-derived EVs 15. Importantly, over the past ten years, miR-223 has attracted great attention as an important regulator in liver pathology 13,16**.** Notably, hepatic miR-223 expression was significantly increased in patients with liver fibrosis and in mice with CCl_4_ or bile duct ligation (BDL)-induced liver fibrosis 17. Calvente *et al.* have demonstrated that neutrophil-derived EVs can transfer miR-223 from neutrophils to hepatic macrophages and covert macrophages into a restorative phenotype that mitigates fibrogenesis 18. In addition, our previous study demonstrated that overexpression of miR-223 ameliorated NASH-related liver fibrosis by inhibiting several targets in hepatocytes 15. However, the functions of miR-223 in HSCs remain largely unknown.

In the current study, we demonstrated that miR-223 played a protective role in liver fibrosis by targeting several genes in hepatocytes and HSCs. The downregulation of miR-223 is accompanied by the activation of HSCs, while neutrophil-derived EVs can transfer miR-223 into HSCs, thus mitigating liver fibrosis.

## Methods and Materials

### Mice

MiR-223^-/-^ (miR-223KO) mice were purchased from the Jackson Laboratory (Bar Harbor, ME). MiR-223KO (miR-223^-/y^) (the miR-223 locus is on the X chromosome) and WT (miR-223^+/y^) littermate controls were generated as previously described 19. All animal experiments were approved by the National Institute on Alcohol Abuse and Alcoholism Animal Care and Use Committee.

For chronic CCl_4_-induced liver fibrosis, mice were injected intraperitoneally with 2 ml/kg body weight of 10% CCl_4_ (Sigma-Aldrich, dissolved in olive oil) twice a week for 4 weeks or 8 weeks. All mice were sacrificed 48 h after the last injection. Serum samples and liver tissues were collected at the indicated time points. For acute CCl_4_-induced acute liver injury, mice were injected intraperitoneally with 2 ml/kg body weight of 10% CCl_4_ and mice were sacrificed 24 h, 48 h or 72h after the injection.

For adenovirus (Ad)-miR-223 injection experiment, mice were treated with CCl_4_ for 4 weeks followed by injection (via the tail vein) of adenovirus expressing mouse miR-223 (Ad-miR-223) (Vector Biolabs, Malvern, PA) or green fluorescence protein (Ad-*Gfp*) (Vector Biolabs) as a control. After another 2 weeks of CCl_4_ treatment, the mice were sacrificed.

### Primary HSC isolation and flow Cytometry Cell Sorter (FACS) sorting

Mice were anesthetized with sodium pentobarbital (30 mg/kg intraperitoneally), the portal vein was cannulated and the liver was perfused with EGTA (ethylene glycol-bis (β-aminoethyl ether)-N,N,N',N'-tetraacetic acid) solution (5.4 mM KCl, 0.44 mM KH_2_PO_4_,140mM NaCl, 0.34 mM Na_2_HPO_4_, 0.5 mM EGTA, and 25 mM Tricine, pH 7.2) and with 0.075% type I collagenase (Worthington, Lakewood, NJ) in Hank's Balanced Salt Solution (Sigma, St. Louis, MO). Liver tissue was further digested with 0.009% collagenase at 37 °C agitation for 20 minutes. Primary hepatocytes were separated after centrifugation at 50 g for 5 minutes and the supernatant was further centrifuged at 400 g for 10 minutes at 4 °C. After centrifuge, the cell pellet was suspended in 11.5% OptiPrep and loaded carefully with Hank's Balanced Salt Solution. After centrifugation at 1500 g for 20 minutes at 4 °C, HSCs were collected from the interface fraction and washed with Hank's Balanced Salt Solution. The finally collected HSCs were cultured in RPMI-1640 medium containing 10% fetal bovine serum, 10% horse serum and penicillin-streptomycin.

For the analysis of *in vivo* miR-223 expression in HSCs, HSCs were isolated from olive oil and CCl_4_-treated mice (4 weeks of CCl_4_ treatment). FACS was conducted following density-gradient centrifugation. HSCs were sorted based on their positive auto fluorescent signals 20.

### Culture of LX-2 cells and miR-223 mimics transfection

Human hepatic stellate cell line LX-2 cells (a kind gift from Dr. Scott Friedman, Mount Sinai) were cultured in DMEM with 10% fetal bovine serum, and penicillin-streptomycin.

MiRNA transfection was performed in primary mouse HSCs and LX-2 cells. Briefly, miR-223 mimics or nonspecific miRNA mimics (NS-miRNA) (Thermo fisher Scientific, Waltham, MA) were transfected into cells at 1 nM using Lipofectamine RNAiMAX transfection reagent (Thermo fisher Scientific, Waltham, MA) following the manufacture's instruction. In some experiments, cells were treated with PDGFBB (PeproTech, Cranbury, NJ; 10 ng/ml) for 20 min after transfection.

### Tissue processing, histological analysis and immunohistochemistry

Formalin fixed liver samples were processed and 4-µm-thick paraffin sections were stained with Sirius Red (collagen/fibrosis) (Sigma, St. Louis, MO). For immunohistochemistry, after heat-induced epitope retrieval, paraffin-embedded sections were incubated in 3% H_2_O_2_, and blocked for another 60 mins in 3% normal serum buffer. Sections were incubated with primary antibodies overnight at 4 °C. Incubation with the secondary antibody against rabbit was performed by SignalStain^®^ Boost IHC Detection Reagent (Cell Signaling Technology, Danvers, MA). DAB Peroxidase Substrate Kit (Vector 2 Laboratories, Inc., Burlingame, CA) were used to visualize the staining according to the manufacturer's instructions. Immunochemistry double staining was performed by using ImmPRESS Duet Double staining Polymer Kit (Vector Laboratories, Burlingame, CA) according to the manufacturer's instructions. The primary antibodies used are listed below: anti-TAZ antibody was purchased from Sigma-Aldrich (Biocare Medical, LLC, Concord, CA); anti-PDGFRα, anti-PDGFRβ, anti-alpha-SMA and anti-Ki67 antibodies were purchased from Cell Signaling Technology (Danvers, MA); anti-IHH was purchased from Novus Biologicals (Centennial, CO) and anti-GLI2 antibody was purchased from Proteintech (Rosemont, IL); anti-myeloperoxidase (MPO) antibody was purchased from Biocare Medical (Concord, CA).The images were taken with an Olympus camera DP72.

### BrdU immunostaining

The rate of cell proliferation was determined by using BrdU immunostaining Kit (BD Biosciences, San Jose, CA) according to the manufacturer's instructions. Primary HSCs were incubated with BrdU (10 μM) for 6 hours before staining. The positively stained HSC nuclei were counted, and the mean values were analyzed.

### Cell counting kit 8 (CCK-8) assay

CCK8 assay was performed using CCK-8 cell counting kit (Vitascientific, MD) according to the manufacturer's instructions. Briefly, cells were seeded into 96-well plates, 10 μl CCK-8 was added to each well and optical density (OD) was measured at 450 nm after incubation for 1.5 hours at 37 °C.

### Co-culture of HSCs and neutrophils

Transwell plates with 0.4 µm pore size polycarbonate membrane inserts (Corning, Glendale, Arizona) were used to set up the co-culture experiments according to the manufacturer's instructions. Primary mouse HSCs were isolated from miR-223KO mice and cultured in the lower chamber for 1 day. Bone marrow neutrophils were isolated by using the mouse neutrophil isolation kit (MiltenyiBiotec, Ashburn, CA) according to the manufacturer's protocol. Neutrophils were added to the upper chamber. After co-culture for 6 hours, the HSCs were collected and subjected to RT-qPCR analysis of miR-223.

### Total RNA isolation and quantitative reverse transcription PCR (RT-qPCR)

Total RNA was extracted from liver tissues or cells using TRIzol reagents (Invitrogen, Carlsbad, CA) according to the manufacturer's instructions. RNA was then reverse transcribed into cDNA using a High-capacity cDNA Reverse Transcription kit (Invitrogen, Carlsbad, CA). The expression levels of mRNA were measured by RT-qPCR by using QuantStudio 6 Flex Real-Time PCR System (Applied Biosystems, Foster City, CA). The mRNA levels of 18s were used as an internal control. The relative expression of mRNA was determined by 2^-∆∆Ct^ method. The primers used for RT-qPCR are listed in Supporting Table S1.

For miRNA detection, total RNA was extracted from liver tissues and cells by using TRIzol reagent (Invitrogen, Carlsbad, CA), and then the mature miRNA strand cDNA was synthesized using TaqMan® MicroRNA Reverse Transcription Kit (Invitrogen, Carlsbad, CA) according to the manufacturer's instructions. MiRNA was amplified by using TaqMan® MicroRNA Assays (Invitrogen) and TaqMan® Universal PCR Master Mix (Invitrogen) according to the manufacturer's instructions. The fold-change for miRNA relative to snoRNA202 or spiked-in cel-miR-39 (Qiagen, Valencia, CA) was calculated by the formula 2^-∆∆Ct^.

### Western blotting

Cells and liver tissues were lysed or homogenized in RIPA buffer containing a cocktail of protease inhibitors (Santa Cruz, CA) according to the manufacturer's instruction. Protein extracts were loaded onto 12% acrylamide gels (Bio-Rad) and transferred onto nitrocellulose membranes. Protein bands were visualized with ECL-chemiluminescent kit (GE Healthcare, Piscataway, NJ). The antibodies against anti-PDGFRα, PDGFRβ, ERK, AKT, phosphorylated ERK (Thr202/Tyr204) and phosphorylated AKT (Ser473) were purchased from Cell Signaling Technology (Danvers, MA). GLI2 and GLI3 antibodies were purchased from Proteintech (Rosemont, IL). The antibodies against β-actin was purchased from Abcam (Cambridge, MA).

### Isolation of EVs and EV uptake assay

EVs were isolated from culture medium of neutrophils or hepatocytes by using ExoQuick-TC solution (System Biosciences, Palo Alto, CA) according to the manufacturer's instructions. For EV uptake assay, the EVs isolated from neutrophils were labeled with DiD red-fluorescent dye (Thermo Fisher Scientific, Inc, Waltham, MA) by incubating with 1 mL DiD red-fluorescent dye solution (1:200 dilution) for 15 min. The labeled EV solution was then centrifuged at 100,000 g for 70 min at 4 °C and re-suspended in PBS and rotated at 4 °C for overnight. The EV fractions were incubated with HSCs for 24 hours and subjected to immunofluorescent staining. PBS was used as a negative control. The images were obtained by using LSM 710 confocal microscope (Zeiss, Thornwood, NY).

### Statistical analysis

Data are expressed as the means ± SEM for each group and were analyzed using GraphPad Prism software (GraphPad Software, La Jolla, CA). To compare values obtained from three or more groups, a one-way ANOVA was used, followed by Tukey post-hoc test. To compare values obtained from two groups, the Student t test was performed. *P* values of <0.05 were considered significant.

## Results

### MiR-223 deletion exacerbates CCl_4_-induced liver fibrosis

**Figure 1:**
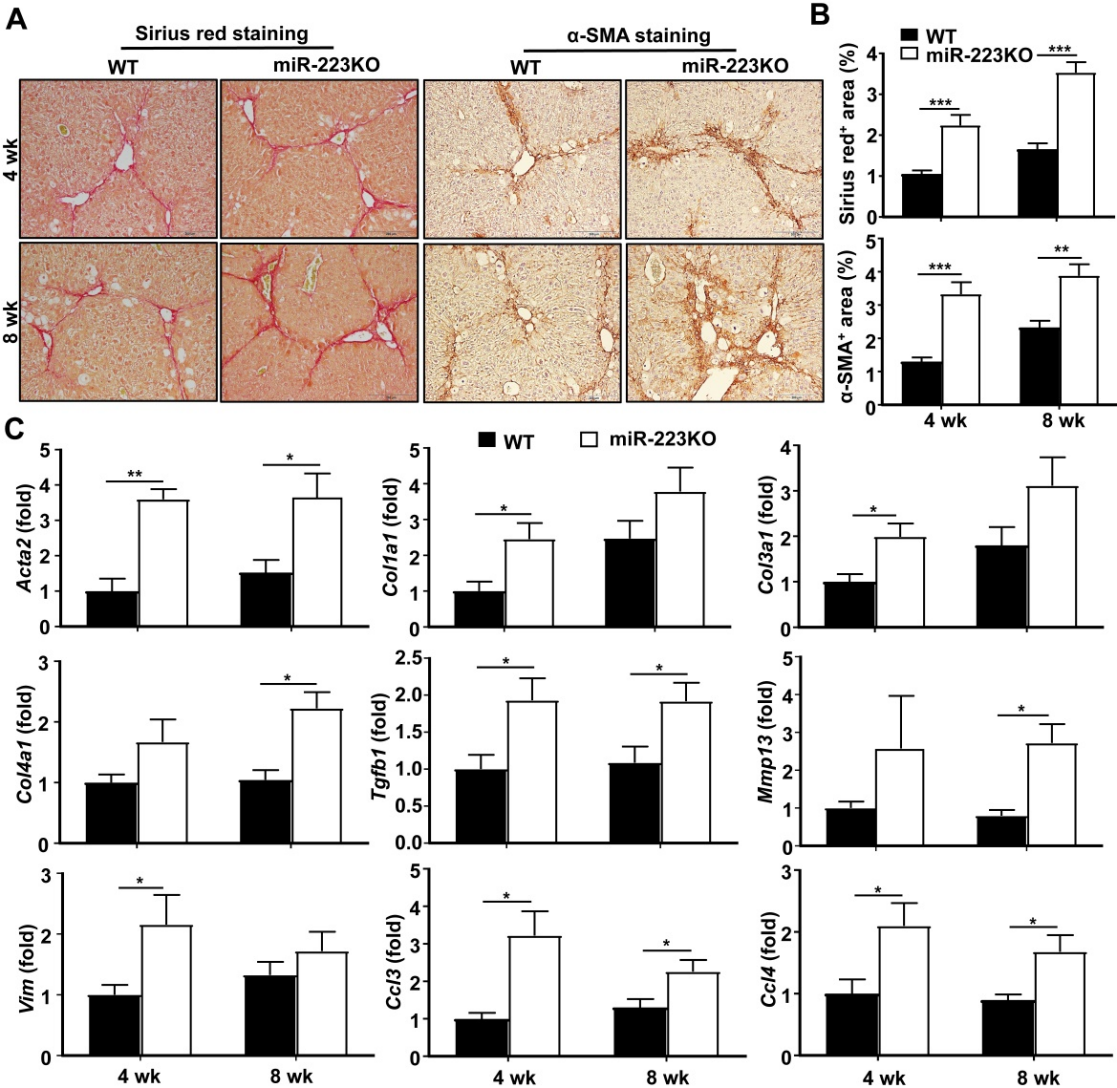
** MiR-223KO mice are more susceptible to CCl_4_-induced liver fibrosis.** WT and miR-223KO mice were treated with CCl_4_ twice a week for 4 weeks or 8 weeks. (A) Representative images of Sirius red and α-SMA staining are shown. (B) Fibrotic area per field was quantified. (C) RT-qPCR analyses of the expression profiles of the genes related to fibrogenesis and inflammation in the liver from WT and miR-223KO mice. Values represent means ± SEM (n=5-8/group). ^*^P< 0.05, ^**^P< 0.01, ^***^P< 0.001.

Our previous work revealed that miR-223KO mice are more susceptible to NASH-related liver fibrosis 19. To further define the functional role of miR-223 in the development of liver fibrosis, miR-223KO mice and their WT littermate control mice were injected with CCl_4_ for 4 weeks or 8 weeks. As demonstrated in Fig. 1A and 1B, miR-223KO mice showed more severe liver fibrosis as evidenced by Sirius red staining and α-SMA staining. Moreover, serum ALT levels were higher in miR-223KO mice compared to WT mice (Supplementary Fig. S1A). Consistently, ALT and AST levels were also higher in miR-223KO mice compared with WT mice in acute liver injury induced by a single injection of CCl_4_ (Supplementary Fig. S1B). Furthermore, hepatic mRNA levels of fibrogenic genes (*Acta2, Col1a1, Col3a1, Col4a1, Tgfb1, Mmp13* and *Vim*) were significantly upregulated in miR-223KO mice compared with WT mice, suggesting that miR-223 deletion accelerated liver fibrosis (Fig. 1C). Increased liver inflammation was further confirmed by the upregulation of *Ccl3* and *Ccl4* in miR-223KO mice, whereas *Ccl2* and* Il6* levels had an increasing trend although no statistical significance was reached between WT and miR-223KO mice (Fig. 1C and Supplementary Fig. S1C).

### MiR-223 attenuates liver fibrosis by inhibiting the TAZ-IHH-GLI2 signaling pathway via the crosstalk between hepatocytes and HSCs

**Figure 2:**
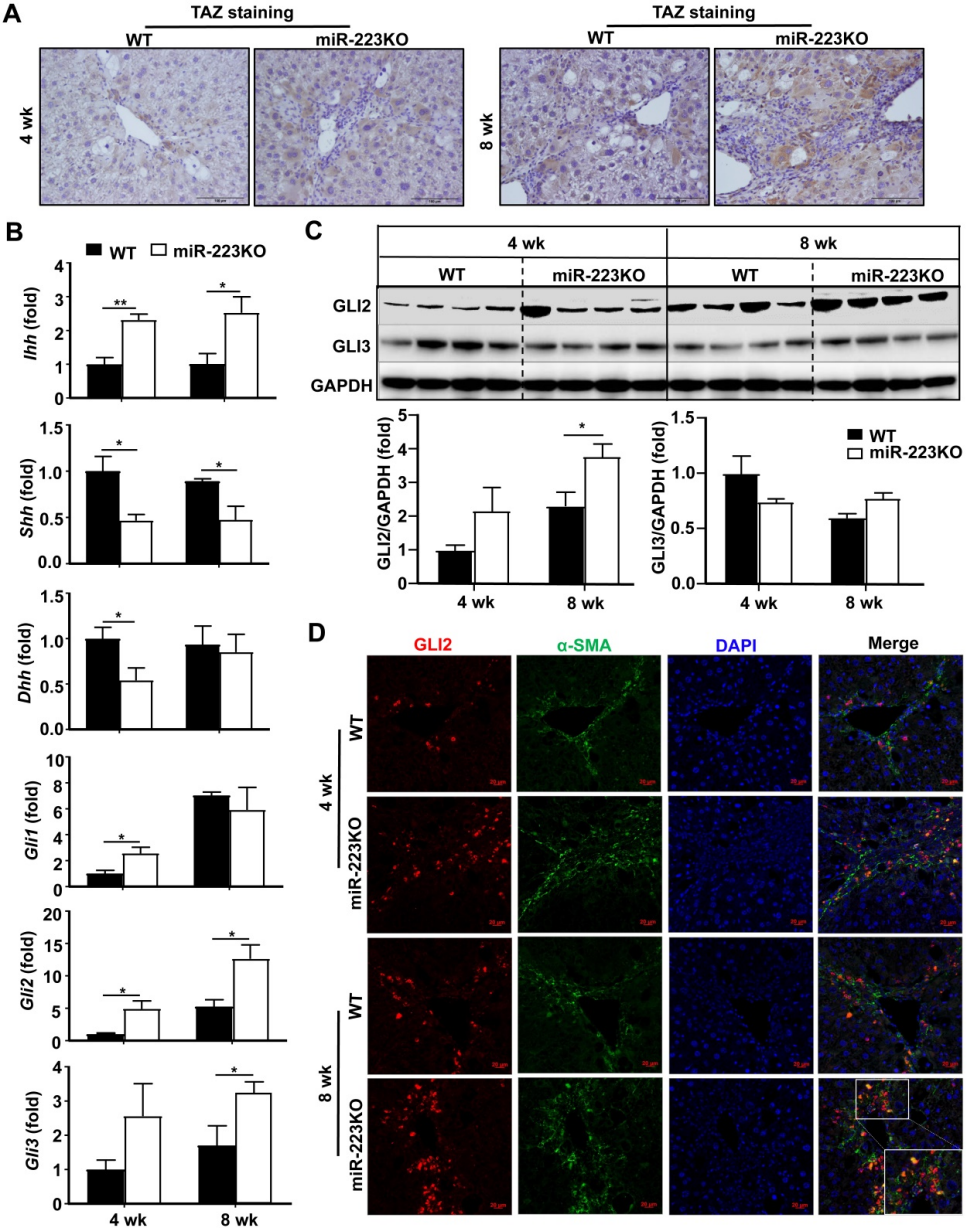
** MiR-223KO mice are more susceptible to CCl_4_-induced liver fibrosis due to enhanced secretion of the TAZ-targeting Indian hedgehog (IHH) by hepatocytes.** WT and miR-223KO mice were treated with CCl_4_ twice a week for 4 weeks or 8 weeks. (A) Representative images of TAZ staining are shown. (B) RT-qPCR analyses of the genes related to Hedgehog signaling pathway in the livers from CCl_4_-treated WT and miR-223KO mice. (C) Liver tissues were subjected to western blot analyses of GLI2 and GLI3 (Upper panel). The blots were quantified (lower panel). (D) Liver tissue samples were subjected to α-SMA and GLI2 immunofluorescence double staining. Representative images of GLI2 (red), α-SMA (green), and nuclei (blue) in the livers from WT and miR-223KO mice are shown. Values represent means ± SEM (n=5-8/group). *P< 0.05, ^**^P< 0.01.

We have previously demonstrated that transcriptional coactivator with PDZ-binding motif (TAZ) is the direct target of miR-223 in hepatocytes in NASH-related liver fibrosis 19. These findings prompted us to investigate whether TAZ in hepatocytes is also the target of miR-223 in CCl_4_-induced liver fibrosis. As shown in Fig. 2A and Supplementary Fig. S2A, TAZ expression in hepatocytes was markedly increased in miR-223KO mice compared to WT mice after chronic CCl_4_ injection. TAZ has been reported to promote liver fibrosis by inducing Indian hedgehog (IHH) in hepatocytes 21, which is a secretory factor that activates the Hedgehog signaling pathway in HSCs and mediates steatosis-to-NASH progression 21. In the presence of Hedgehog ligand, including IHH, Sonic hedgehog (SHH) and Desert hedgehog (DHH), the signal transducer Smoothened is activated, which promotes the accumulation and nuclear translocation of transcription factor GLI family including GLI1, GLI2, and GLI3, finally activating HSCs 1,22. To further clarify whether miR-223 attenuates liver fibrosis via TAZ-Hedgehog pathway in CCl_4_-induced liver fibrosis, we analyzed the expression of Hedgehog signaling-related factors in the liver of WT and miR-223KO mice. Indeed, as illustrated in Fig. 2B, miR-223KO mice exhibited higher hepatic mRNA levels of *Ihh* than WT mice; however, hepatic Shh and Dhh mRNA levels were downregulated in miR-223KO mice compared with WT mice. Other hedgehog signaling-related gene (e.g *Smo*, *Ptch1*) levels showed no significant difference between WT and miR-223KO mice (Supplementary Fig. S2B). Immunohistochemistry staining also revealed that the greater hepatic IHH expression in miR-223KO mice than that in WT mice after chronic CCl4 treatment (Supplementary Fig. S2C). Additionally, the expression of transcription factor GLI family especially *Gli2* was remarkably elevated in miR-223KO mice. Elevation of GLI2 protein in miR-223KO mice was also observed by western blot analysis (Fig. 2C). To further determine which cell type expressed GLI2 in the liver, we performed α-SMA and GLI2 immunofluorescence double staining in the livers of WT and miR-223KO mice. Notably, GLI2 expression was highly elevated in the fibrotic area with co-staining of α-SMA in miR-223KO mice compared with WT mice (Fig. 2D). Taken together, these results strongly suggest that miR-223 inhibits paracrine IHH production by targeting TAZ in hepatocytes, leading to inhibition of Hedgehog signaling pathway in HSCs.

### *Gli2* is a target of miR-223 in HSCs

**Figure 3:**
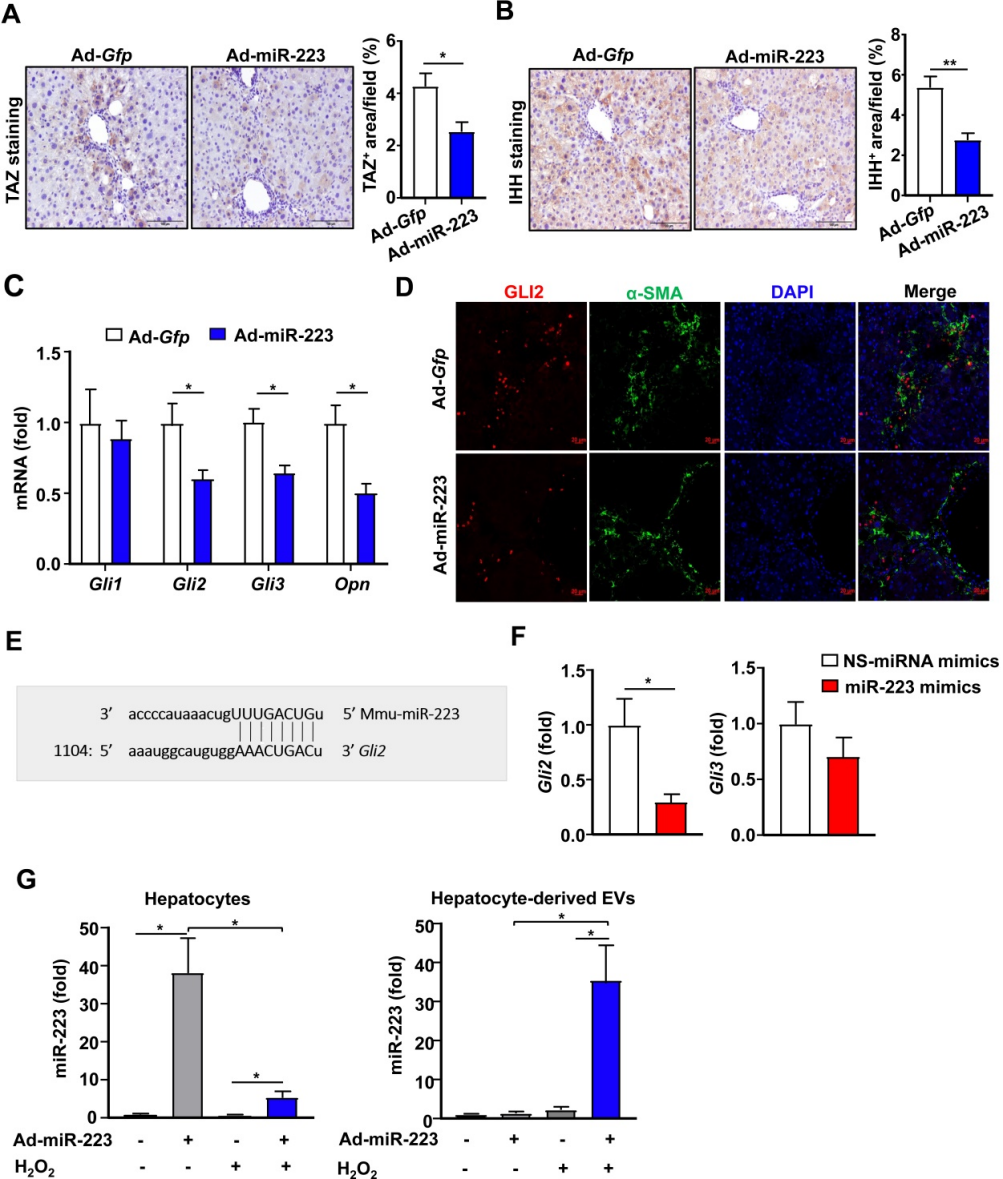
** Overexpression of miR-223 attenuates TAZ and GLI2 expression in the liver and directly inhibits *Gli2* in HSCs.** (A-D) C57BL/6J mice were injected with CCl_4_ for 6 weeks and were given intravenous injection of Ad-*Gfp* or Ad-miR-223 at the end of the 4^th^ week. (n=6/group). The liver tissues were collected at the 6^th^ week. (A-B) Representative images of TAZ staining and IHH staining are shown. Quantification of the TAZ^+^ and IHH^+^ area per field was performed and is shown. (C) RT-qPCR analyses of *Gli2*, *Gli3* and* Opn* in liver tissues. (D) Representative images of GLI2 (red), α-SMA (green), and nuclei (blue) in the livers are shown. (E) Using a miRNA database (http://www.targetscan.org/), putative binding sites of miR-223 were predicted in the 3'-UTR of *Gli2* mRNA in mice. The dashed line represents complementary base pairs between miR-223 and *Gli2* 3'-UTR. (F) Primary mouse HSCs were transfected with miR-223 mimics and nonspecific miRNA mimics (NS) for 24 hours. The mRNA levels of *Gli2* and *Gli3* were analyzed by RT-qPCR (G) Primary hepatocytes were transfected with Ad-miR-223 for 24 hours followed by H_2_O_2_ (500 µM, 5h) treatment. The expression of miR-223 in hepatocytes and hepatocyte-derived EVs was analyzed by RT-qPCR. Values represent means ± SEM. In panels A-D, n=6/group; Panels F-G are from three independent experiments. *P< 0.05.

The above data suggest that the increased GLI2 expression in the HSCs from miR-223KO mice is due to the enhanced stimulation of IHH produced by hepatocytes. Next we investigated the effect of miR-223 overexpression in hepatocytes on CCl_4_-induced liver fibrosis* in vivo*. Mice were injected with CCl_4_ for 4 weeks followed by *Gfp-* or miR-223 expressing adenovirus injection for 2 weeks. As we previously reported, overexpression of miR-223 led to decreased fibrogenic gene expression and attenuated CCl_4_-induced liver fibrosis 23. In the current study, we demonstrated that TAZ and IHH protein levels in hepatocytes were markedly decreased in mice injected with miR-223-expressing adenovirus (Ad-miR-223) (Fig. 3A and 3B). In addition, hepatic expressions of* Gli2*,* Gli3* and IHH target gene, osteopontin (*Opn*) 24, were downregulated after administration of Ad-miR-223 (Fig. 3C). Consistently, immunofluorescence staining revealed that injection of Ad-miR-223 reduced GLI2 expression in HSCs in CCl_4_-induced liver fibrosis (Fig. 3D).

Next, we wondered whether GLI2 can be directly regulated by miR-223 in HSCs. To verify this hypothesis, we conducted bioinformatic analysis by using targetscan (http://www.targetscan.org/). As a result, miR-223 was predicted to directly bind the 3'-UTR of *Gli2* (Fig. 3E). To determine the function of miR-223 in regulating GLI2 expression, we overexpressed miR-223 in primary mouse HSCs via the transfection of miR-223 mimics. As shown in Fig. 3F, overexpression of miR-223 significantly suppressed *Gli2* but not *Gli3* expression in primary HSCs.

Given that adenovirus are vehicles which mainly deliver genes to hepatocytes, we speculated that overexpressed miR-223 in hepatocyte can be transferred into HSCs by hepatocyte-derived EVs. To address this issue, we examined the expression of miR-223 in hepatocyte-derived EVs after transduction of Ad-miR-223 *in vitro*. Since oxidative stress plays a vital role in CCl_4_-induced toxicity, we treated hepatocytes with hydrogen peroxide (H_2_O_2_), which is an inducer of oxidative stress *in vitro* 25. As shown in Fig. 3G, miR-223 expression in hepatocytes was dramatically elevated after treatment with Ad-miR-223; however, after treatment with H_2_O_2_, such miR-223 expression in hepatocytes was markedly reduced while miR-223 express was highly elevated in hepatocyte-derived EVs. These data suggest that oxidative stress can stimulate hepatocytes to release miR-223 enriched EVs.

### MiR-223 ameliorates liver fibrosis by targeting PDGFRα/β in HSCs

**Figure 4:**
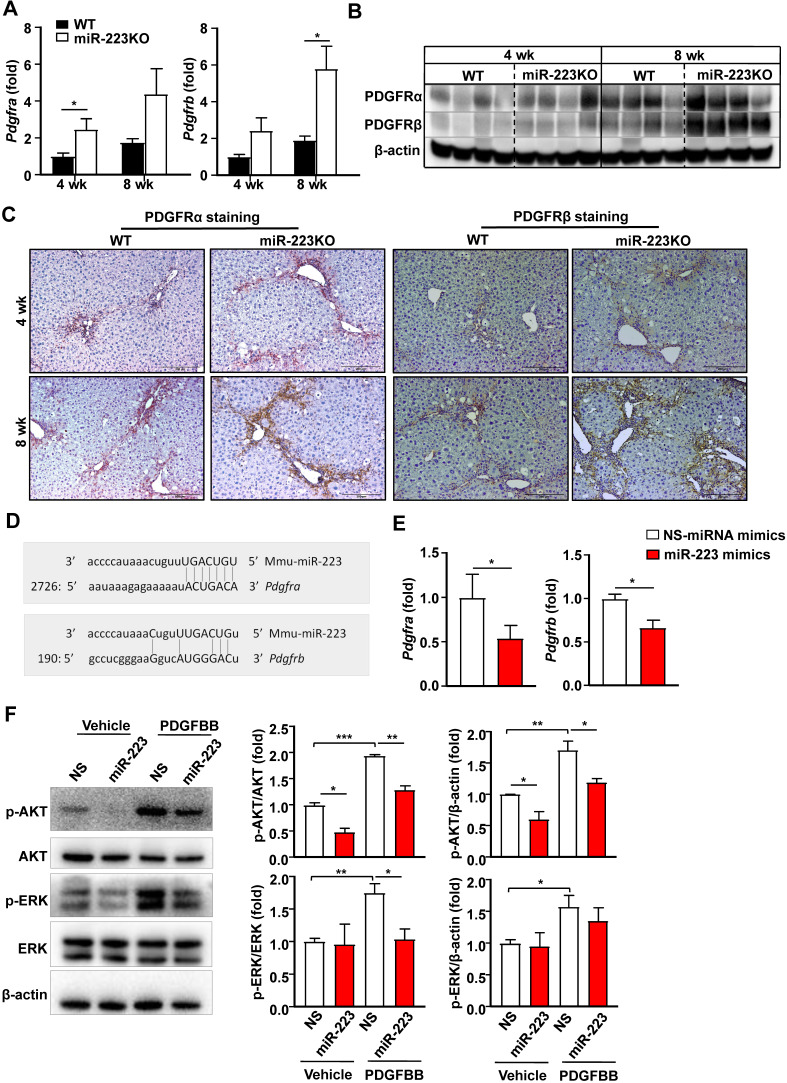
** MiR-223 inhibits PDGF signaling pathway in HSCs by targeting *Pdgfra* and *Pdgfrb*.** (A-C) WT and miR-223KO mice were treated with CCl_4_ twice a week for 4 weeks or 8 weeks. (A) RT-qPCR analyses of *Pdgfra* and *Pdgfrb*. (B) Liver tissues were subjected to western blot analyses of PDGFRα and PDGFRβ. (C) Representative images of PDGFRα and PDGFRβ staining are shown. (D) Using a miRNA database (http://www.targetscan.org/), putative binding sites of miR-223 were predicted in the 3'-UTR of mouse *Pdgfra* and *Pdbgrb* mRNAs. (E) Primary mouse HSCs were transfected with miR-223 mimics and nonspecific miRNA mimics (NS), and the mRNA levels of *Pdgfra* and *Pdgfrb* were detected by RT-qPCR analyses. (F) Primary mouse HSCs were transfected with miR-223 mimics and nonspecific miRNA mimics (NS) for 24 hours followed by treatment with vehicle or PDGFBB (10 ng/ml) for 20 min. Total cell lysates were subjected to western blot analyses of PDGF signaling pathway. The blots were quantified (right panel). Values represent means ± SEM. In panels A-C, n=5-8/group; Panels E-F from three independent experiments. ^*^P< 0.05, ^**^P< 0.01,^ ***^P< 0.001.

PDGF signaling plays a critical role in promoting HSC proliferation and migration 26,27. MiR-223 has been reported to directly target PDGF receptor β (PDGFRβ) in vascular smooth muscle cells 28. We speculated that miR-233 may interfere with the PDGF signaling pathway by modulating PDGFR expression in HSCs. To address this hypothesis, we first analyzed the expression of PDGFRα and PDGFRβ in the livers of chronic CCl_4_-treated WT and miR-223KO mice. As illustrated in Fig. 4A, B and Supplementary Fig. S3A, hepatic PDGF receptor α (PDGFRα) and PDGFRβ mRNA and protein levels were significantly elevated in chronic CCl_4_-treated miR-223KO mice compared to chronic CCl_4_-treated WT mice. Immunohistochemistry staining further identified a greater expression of PDGFRα and PDGFRβ in the fibrotic regions of chronic CCl_4_-treated miR-223KO mice than in WT mice (Fig. 4C and Supplementary Fig. S3B). To further investigate whether miR-223 can regulate PDGFRα/β expression, we performed bioinformatics analysis and identified the binding site for miR-223 in the 3'-UTR of *Pdgfra* and *Pdgfrb* (Fig. 4D). The direct target of PDGFRα and PDGFRβ by miR-223 was further supported by RT-qPCR data showing that overexpression of miR-223 significantly downregulated the expressions of PDGFRα and PDGFRβ in HSCs (Fig. 4E). PDGF-BB, a ligand for PDGF signaling, binds to PDGF receptors and induces phosphorylation of ERK and AKT, thereby leading to HSC proliferation, activation and migration 3. As illustrated in Fig. 4F, PDGF-BB treatment induced the phosphorylation of ERK and AKT, such phosphorylation was blocked by overexpression of miR-223 in primary HSCs. These findings support the notion that miR-223 inhibits the activation of PDGF signaling pathway in HSCs by targeting PDGFRα/β.

### MiR-223 directly inhibits HSC activation and proliferation

**Figure 5:**
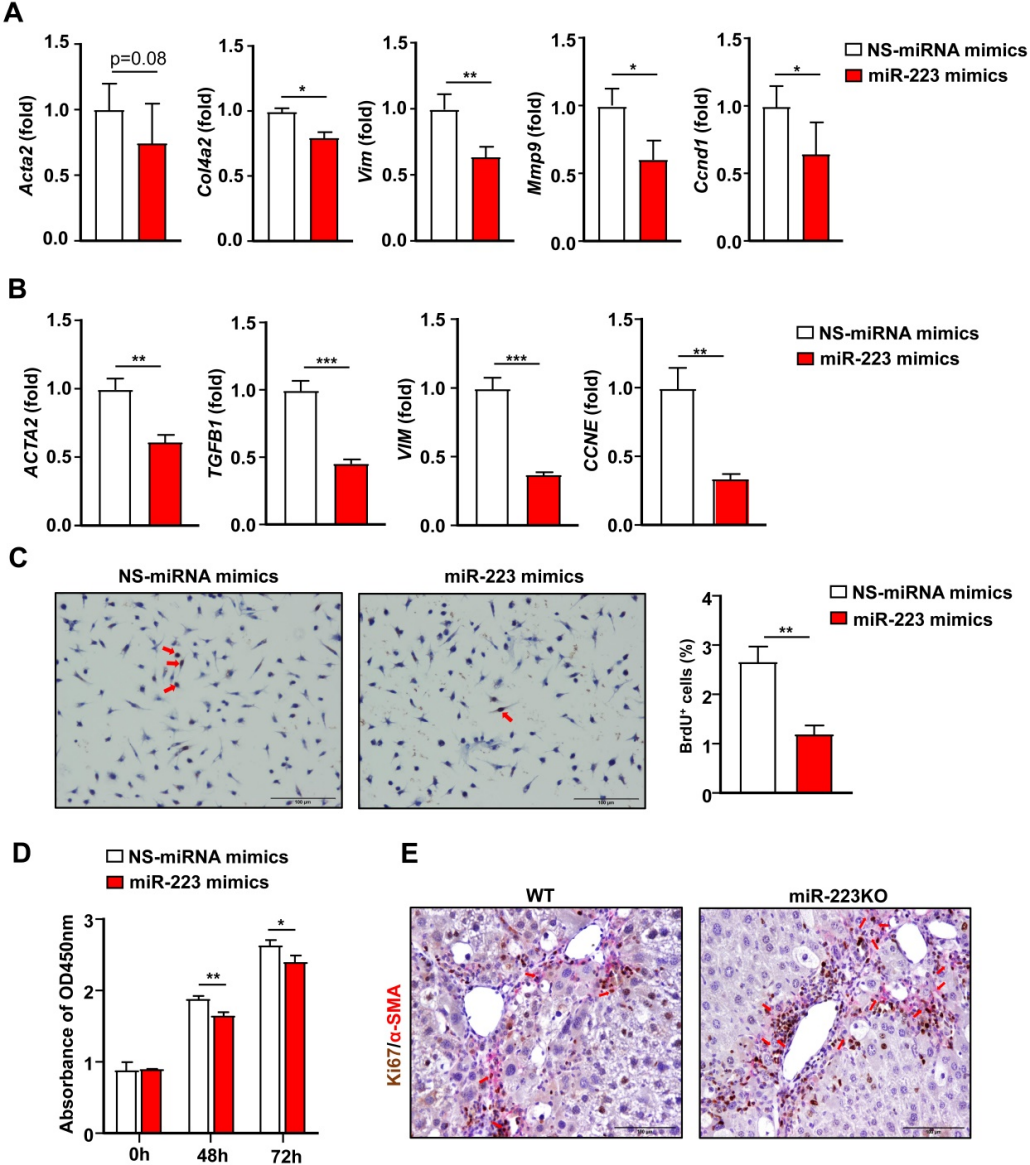
** Overexpression of miR-223 suppresses HSC activation and proliferation.** (A) Primary mouse HSCs were transfected with miR-223 mimics and nonspecific miRNA mimics (NS) for 24 hours. The expression of the genes related to fibrogenesis and cell proliferation was analyzed by RT-qPCR. (B) Human HSC cell line LX-2 cells were transfected with miR-223 mimics and nonspecific miRNA mimics (NS) for 48 hours. The expression of genes involved in fibrogenesis and cell proliferation was analyzed by RT-qPCR. (C) Primary mouse HSCs were transfected with miR-223 mimics and nonspecific miRNA mimics (NS) for 24 hours. Cells were incubated with BrdU (10 uM) for 6 hours before staining. Representative images of BrdU staining are shown in the left panel. The number of BrdU^+^ HSCs was counted in the right panel. (D) LX-2 cells were transfected with miR-223 mimics and nonspecific miRNA mimics (NS) for 48 hours and 72 hours followed by CCK8 assay. (E) Liver tissue samples from 8-week CCl_4_-treated WT and miR-223KO mice were subjected to α-SMA and Ki67 immunofluorescence staining. Representative images of α-SMA (pink) and Ki67 (brown) in the liver from WT and miR-223KO mice are shown. Values represent means ± SEM. Panels A-D from three independent experiments. In panel E, n=5-8/group. *P< 0.05, ^**^P< 0.01, ^***^P< 0.001.

Activation of HSCs is the central event in liver fibrosis 3, thus we focused on the effects of miR-223 on HSCs for the rest of our study. We have previously demonstrated that primary HSCs from miR-223KO mice expressed higher levels of fibrotic genes 19, indicating that miR-223 deficiency promotes fibrogenesis in HSCs. In addition, the above data also show that miR-223 directly inhibits GLI2, PDGFRα and PDGFRβ in HSCs. Collectively, this prompted us to hypothesize that miR-223 may directly inhibit HSC activation. To test this hypothesis, we overexpressed miR-223 in primary mouse HSCs and human HSC cell line LX-2 cells via the transfection of miR-223 mimics. As illustrated in Fig. 5A, overexpression of miR-223 downregulated the expression of* Acta2, Col4a2*, *Vim* and *Mmp9*. Consistently, in LX-2 cells, overexpression of miR-223 leads to decreased expression of* ACTA2*, *TGFB1*, and *VIM* (Fig. 5B). Interestingly, the cell cycle genes *Ccnd1* and *CCNE* were also downregulated by miR-223 in primary HSCs and LX-2 cells, respectively (Fig. 5A and 5B). These results promoted us to determine the function of miR-223 in the proliferation of HSCs. We performed BrdU staining in primary HSCs and observed the reduced number of BrdU positive cells in HSCs after overexpression of miR-223 (Fig. 5C). Besides, CCK-8 assay further revealed that overexpression of miR-223 significantly suppressed the proliferation of LX-2 cells (Fig. 5D). To further confirm the role of miR-223 in HSC proliferation *in vivo*, we performed α-SMA and Ki67 immunofluorescence double staining in the livers of WT and miR-223KO mice after 8 weeks CCl_4_ injection. Notably, more Ki67 and α-SMA double positive cells were detected in miR-223KO mice compared with WT mice, suggesting that miR-223 deletion enhances the proliferation of HSCs in liver fibrosis (Fig. 5E).

### Regulation of miR-223 expression in HSCs during the progression of liver fibrosis

**Figure 6:**
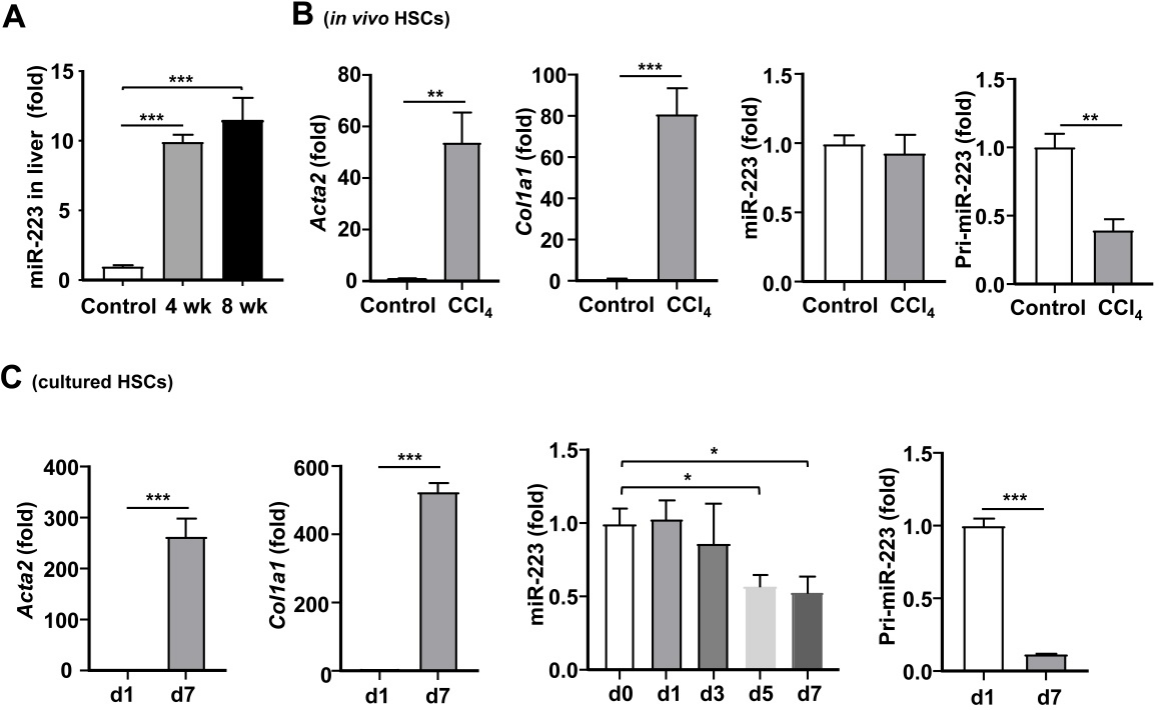
** Regulation of miR-223 expression *in vivo* and *in vitro* during HSC activation.** (A) C57BL/6J mice were injected with CCl_4_ for 4 weeks or 8 weeks. Hepatic miR-223 was measured by RT-qPCR. (B) C57BL/6J mice were injected with CCl_4_ for 4 weeks. Primary HSCs were isolated from CCl_4_-treated mice and control mice. *Acta2*, *Col1a1*, miR-223 and pri-223 expression levels were measured by RT-qPCR. (C) Primary HSCs were isolated and cultured for 1 day, 3 days, 5 days and 7 days, the expression of *Acta2*, *Col1a1*, miR-223 and pri-miR-223 was analyzed by RT-qPCR at indicated time points. Values represent means ± SEM. In panel A, n=5-8/group. Panels B-C from three independent experiments. *P< 0.05, **P< 0.01, ***P< 0.001.

Our previous data show that hepatic miR-223 expression is increased in a NASH-related fibrosis mouse model induced by high fat diet feeding 19. Therefore, we hypothesized that miR-223 expression might be altered during the pathogenesis of liver fibrosis. Similar to the result of NASH-related liver fibrosis 19, hepatic miR-223 expression was significantly increased after chronic CCl_4_ treatment (Fig. 6A). To determine whether miR-223 expression in HSCs was altered *in vivo* during fibrosis, we isolated primary HSCs from mice treated with and without CCl_4_, followed by analysis of miR-223 expression. As demonstrated in Fig. 6B, as expected, *Acta2* and *Col1a1* mRNA levels were dramatically elevated in activated HSCs from CCl_4_-treated mice compared with quiescent HSCs from control mice; however, miR-223 expression was comparable between activated HSCs and quiescent HSCs. To our surprise, pri-miR-223, which is the precursor of miR-223 29, was significantly decreased in activated HSCs from CCl_4_-treated mice compared to quiescent HSCs from control mice.

To further examine the effect of HSC activation on miR-223 expression, we examined the expression of miR-223 at different time points during HSC activation *in vitro.* As illustrated in Fig. 6C, compared to freshly isolated quiescent HSCs, miR-223 expression was decreased in activated HSCs on day 5 and day 7. The expression of *Acta2* and *Col1a1* mRNAs was highly elevated in activated HSCs, confirming the HSC activation. Furthermore, pri-miR-223 expression was also reduced in activated HSCs compared to quiescent HSCs.

### Activated HSCs take up neutrophil-derived EVs, thus elevating miR-223

The above data show that the biosynthesis of miR-223 is decreased in activated HSCs *in vivo* and *in vitro*, but mature miR-223 remains unchanged in activated HSCs *in vivo,* suggesting the possibility that other types of cells may compensate the reduction of miR-223 levels in activated HSCs *in vivo* by transferring miR-223.

**Figure 7:**
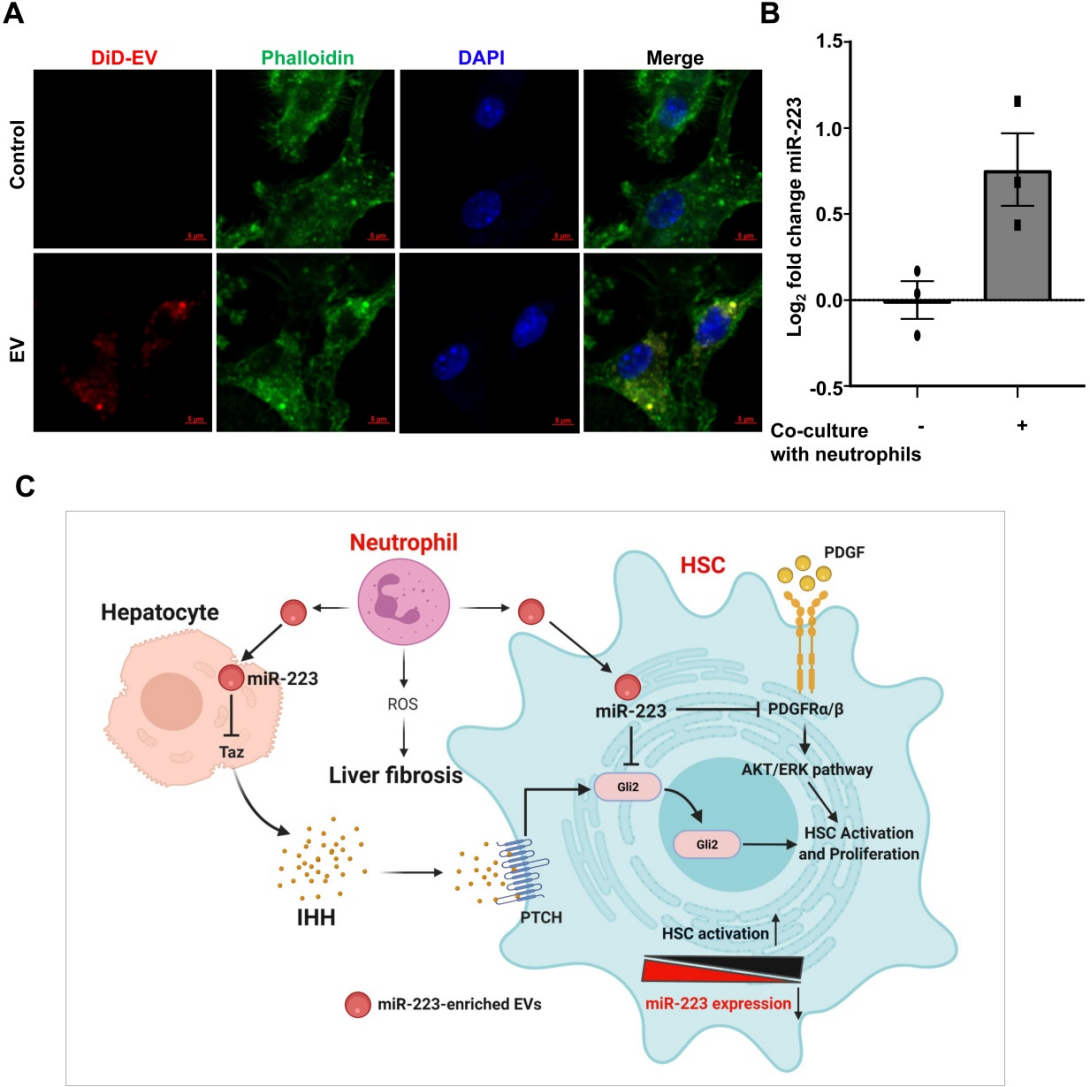
** Activated HSCs take up neutrophil-derived EVs, thus elevating miR-223.** (A) EVs derived from neutrophils were fluorescently labeled with the cell membrane dye DiD prior to its incubation with HSCs. The HSCs were incubated with fluorescently labeled-EVs for 24 hours. Representative images of DiD immunofluorescence (red), F-actin (green) and nuclei (blue) are shown. (B) Primary HSCs were isolated from miR-223KO mice. The expression of miR-223 was measured in HSCs after co-culture with bone marrow neutrophils for 6 hours. Values represent means ± SEM from three independent experiments. (C) Schematic representation of the mechanism by which miR-223 inhibits HSC activation and proliferation. MiR-223 protects against liver fibrosis by regulating multiple targets in different hepatic cells. In hepatocytes, miR-223 inhibits TAZ expression, leading to decreased IHH secretion from hepatocytes, which serves as the ligand for Hedgehog signaling in HSCs. In HSCs, miR-223 directly suppresses *Gli2* and *Pdgfra/b* expression and eventually suppresses HSC activation and proliferation. During the activation of HSCs, miR-223 expression is decreased, which accelerates HSC activation. Neutrophils can deliver miR-223 into HSCs by EVs, which can compensate the decreased miR-223 biosynthesis in HSCs *in vivo*, thereby inhibiting liver fibrosis. (Image created with BioRender.com).

In chronic CCl_4_-induced liver fibrosis model, CCl_4_ injection induces hepatocyte death, followed by infiltration of inflammatory cells, such as neutrophils 30. We detected significant neutrophil infiltration in fibrotic areas after chronic CCl_4_ treatment as evidenced by MPO (a neutrophil marker) staining in Supplementary Fig. S4. Since neutrophils express miR-223 at very high levels and can transfer miR-223 into hepatocytes 15 and macrophages 9, we hypothesized that during liver fibrosis, miR-223 can be transferred from neighboring neutrophils into HSCs. To address this question, we tested whether HSCs can take up neutrophil-derived miR-223 enriched EVs. As illustrated in Fig. 7A, after co-cultured with fluorescence (DiD)-labelled neutrophil-derived EVs, HSCs had significant amount of fluorescence staining, suggesting that HSCs can take up neutrophil-derived EVs. Furthermore, we co-cultured WT neutrophils with miR-223 KO HSCs isolated from miR-223KO mice, and observed detectable miR-223 expression in HSCs (Fig. 7B). Since miR-223 deficient HSCs do not have miR-223, we concluded that the detected miR-223 was due to its transfer from neutrophils into HSCs.

## Discussion

To our knowledge, the current study demonstrated for the first time that miR-223 directly inhibits HSC activation and proliferation by targeting *Gli2* and *Pdgfra/b*, thus ameliorating liver fibrosis. Furthermore, results from our mechanistic studies suggest that pri-miR-223 and miR-223 expression are downregulated during HSC activation *in vitro*, and such downregulation may accelerate HSC activation. However, despite of pri-miR-223 downregulation, miR-223 expression was not decreased in activated HSCs *in vivo* from fibrotic livers. *In vitro* co-culture experiments demonstrated that HSCs can take up neutrophil-derived miR-223-enriched EVs. We have integrated all of these findings together into a model depicting how miR-223 restricts liver fibrosis via the potential crosstalk of several types of cells in the liver (Fig. 7C).

The anti-fibrotic role of miR-223 has been reported in NASH-related fibrosis 15,19,31. However, the functional roles of miR-223 in CCl_4_-induced liver fibrosis and in HSCs have not been fully illustrated. Our data clearly revealed that miR-223KO mice had greater liver fibrosis as evidenced by stronger Sirius red and α-SMA immunostaining and increased fibrogenesis genes expression, suggesting that miR-223 plays a protective role in CCl_4_-induced liver fibrosis. Our finding is in line with the previous report by Calvente *et al.* which demonstrated that miR-223 deficiency impaired the spontaneous resolution of liver fibrosis caused by CCl_4_ administration 9. However, Schueller *et al.* reported no differences in the degree of liver injury and fibrosis between WT and miR-223KO mice in the chronic CCl_4_ mouse model 17. The reasons for the discrepancy among these reports may be due to the difference in animal facility, the source of mice, different WT control mice used, and different doses of CCl_4_ treatment. In our current study, we used littermate WT mice as the control group; while non-littermate WT control mice were used in other studies 17.

Till date, various targets of miR-223 have been identified in hepatocytes, neutrophils and macrophages 13; however, the targets of miR-223 in HSCs have not been explored. In the current study, we have identified several targets of miR-223 in HSCs. First, we demonstrated that *Gli2* is a direct target of miR-223 in HSCs *in vivo* and *in vitro*. GLI2, which is a transcriptional activator of Hh signaling pathway 22, was significantly elevated in miR-223KO mice and downregulated by Ad-miR-223 administration after CCl_4_ challenge. Inhibition of GLI2 may contribute to the anti-fibrotic functions of miR-223. Second, we also found that *Pdgfra* and *Pdgfrb* are targets of miR-223 in HSCs and overexpression of miR-223 in HSCs blunted PDGF downstream ERK and AKT signaling, thus inhibiting HSC proliferation. PDGR signaling is one of the most important pathways which regulates activation and proliferation of HSCs during hepatic fibrosis 26,27. It has been reported that PDGFRα promotes HSC proliferation and migration 26,32, while PDGFRβ induces the activation and transdifferentiation of HSCs into myofibroblasts 33. Taken together, miR-223 suppresses the activation and proliferation of HSCs by regulating Hh and PDGF signaling pathway.

In addition to the targets of miR-223 in HSCs, our data also suggest that miR-223 limits liver fibrosis partially by targeting TAZ in hepatocytes. TAZ has been implicated in promoting liver fibrosis, particularly in the steatosis-to-NASH conversion via the induction of IHH in hepatocytes, a secretory factor that activates HSCs 21. Of note, overexpression of miR-223 in hepatocytes ameliorates CCl_4_-induced liver fibrosis along with the hepatic downregulation of TAZ and IHH. However, the mechanism by which hepatic *Shh* and *Dhh* mRNA levels are decreased in miR-223KO mice compared with WT mice after chronic CCl_4_ injection remains unclear. Further studies are needed to investigate whether SHH and DHH can be regulated by miR-223. Collectively, miR-223 ameliorates liver fibrosis by regulating multiple targets in HSCs and hepatocytes.

In mouse models of liver fibrosis induced by BDL or chronic CCl_4_ injection, hepatic miR-223 levels were found to be elevated 17. In line with previous reports, we also observed the increased hepatic miR-223 expression after chronic CCl_4_ treatment. One of the major reasons for elevated miR-223 expression in the fibrotic lives is probably due to the infiltration of neutrophils that express much higher miR-223 than hepatocytes 34. Interestingly, miR-223 expression was markedly downregulated in activated HSCs *in vitro* but was not reduced in activated HSCs *in vivo* from CCl_4_-induced fibrotic livers despite of downregulation of pri-miR-223 in both *in vitro* and *in vivo* activated HSCs. Levels of mature miR-223 in the cells are controlled by biogenesis, secretion and uptake of miR-223 via the EVs. Because the biogenesis (pri-miR-223) was reduced, the unchanged miR-223 levels in activated HSCs *in vivo* were probably due to reduced secretion and/or increased uptake of miR-223. At the present, we do not have data whether secretion of miR-223 was reduced in activated HSCs, however, several lines of evidence suggest that activated HSCs may have increased uptake of miR-223 in the fibrotic livers *in vivo*. First, neutrophils, which expressed miR-223 at the highest levels among all types of cells, are accumulated in the fibrotic regions, and contact activated HSCs. Second, previous studies have reported that neutrophils can transfer miR-223 into macrophages 9 and hepatocytes 15 via the EVs. In the current study, we found that neutrophil-derived EVs can be taken up by HSCs, leading to elevated miR-223 levels in HSCs. The mechanism of EV uptake by HSCs is a challenging question. Several endocytic pathways have been implicated in EV uptake, including endocytosis, micropinocytosis, phagocytosis, and lipid raft-mediated internalization 35. Different cell types likely have different underlying mechanisms for EV uptake. For example, low-density lipoprotein receptor on hepatocytes has been shown to promote selective uptake of miR-223-enriched EVs from neutrophils in obese mice 15. Besides, several stimuli such as TGFβ may also be involved in regulating EV uptake 36. Further studies are warranted to elucidate the mechanisms of EV uptake by HSCs in the pathogenesis of liver fibrosis.

Hepatic neutrophil infiltration is a hallmark for liver inflammation in various types of liver diseases. However, the mechanisms by which neutrophils contribute to liver fibrosis remain obscure. One previous study showed that impaired neutrophil recruitment does not impact on CCl_4_-induced liver fibrosis 37. While Calvente et al reported that neutrophil depletion impaired resolution from both CCl_4_- and MCD-induced liver fibrosis 9. To better understand the function of neutrophils in liver fibrosis, some studies have focused on the communication between neutrophils and HSCs 6. Zhou et al. demonstrated that activated HSCs prolong the survival of neutrophils, which further promotes liver fibrosis via the production of reactive oxygen species (ROS) 6. However, our data suggest a protective role of neutrophils in liver fibrosis by delivering exosomal miR-223 into HSCs, and reveal an important mechanism responsible for neutrophils in regulating liver fibrosis, which will help understand the complex roles of neutrophils in the progression from chronic liver inflammation to fibrosis. Further studies are needed to clarify the specific functions of miR-223 in neutrophils in liver fibrosis by using neutrophil-specific miR-223 knockout mice.

Due to an important role of miR-223 in inhibiting HSC activation and liver fibrosis, elevation of miR-223 levels may open new avenues for the treatment of liver fibrosis in the future. Indeed, Calvente et al have reported the therapeutic effect of the synthetic miR-223 analog miR-223-3p in the progression of chronic inflammation and fibrosis in NASH 31. Restoration of hepatic miR-223 ameliorates steatosis and fibrosis in HFD-fed *Ldlr* KO mice 15. In addition, the therapeutic effect of adenovirus-mediated overexpression of miR-223 was also recently demonstrated in liver fibrosis induced by chronic CCl_4_ treatment 23. In the current study, we demonstrated that overexpression of miR-223 in hepatocytes can also ameliorate CCl_4-_induced liver fibrosis by secreting TAZ-targeting IHH and by releasing miR-223-enriched EVs. Since HSCs are in close contact with hepatocytes *in vivo*, we predicted that adenovirus-mediated overexpression of miR-223 in hepatocytes may be an effective approach to treat liver fibrosis not only via the direct inhibition of fibrogenic genes in hepatocytes but also via the production of miR-223-enriched EVs that can be transferred into HSCs. Finally, due to the high stability and rapid biodistribution of EVs in the liver 38, it will be interesting to explore the therapeutic effect of miR-223-loaded EVs in liver fibrosis.

## Supplementary Material

Supplementary figures and tables.

## Abbreviations

ALT: alanine aminotransferase
BDL: bile duct ligation
CCl_4_: carbon tetrachloride
DHH: Desert hedgehog
ECM: extracellular matrix
EVs: extracellular vesicles
GLI2: GLI Family Zinc Finger 2
H_2_O_2_: hydrogen peroxide
Hh: Hedgehog
miRNAs: microRNAs
HSCs: hepatic stellate cells
KO: knockout
MPO: myeloperoxidase
NASH: nonalcoholic steatohepatitis
Opn: osteopontin
PDGF: platelet-derived growth factor
ROS: reactive oxygen species
RT-qPCR: reverse transcription quantitative PCR
SHH: Sonic hedgehog
TAZ: PDZ-binding motif
TGFβ: transforming growth factor β
UTRs: untranslated regions
WT: wild-type
