# Supplementary material for: MicroRNA-223 restricts liver fibrosis by inhibiting the TAZ-IHH-GLI2 and PDGF signaling pathways via the crosstalk of multiple liver cell types
Source: Int J Biol Sci. 2021 Mar 11;17(4):1153–67. doi: 10.7150/ijbs.58365 (PMC8040312; doi:10.7150/ijbs.58365)
Supplement: Supplementary file 1 — Supplementary figures and tables. [file ijbsv17p1153s1.pdf]

MicroRNA-223 restricts liver fibrosis by inhibiting the TAZ-IHH-GLI2 and PDGF signaling pathways via the crosstalk of multiple liver cell types

Xiaolin Wang[1,2], Wonhyo Seo[1], Seol Hee Park[1], Yaojie Fu[1], Seonghwan Hwang[1], Robim M. Rodrigues[1], Dechun Feng[1], Bin Gao[1]*, Yong He[1]*

Supporting Materials

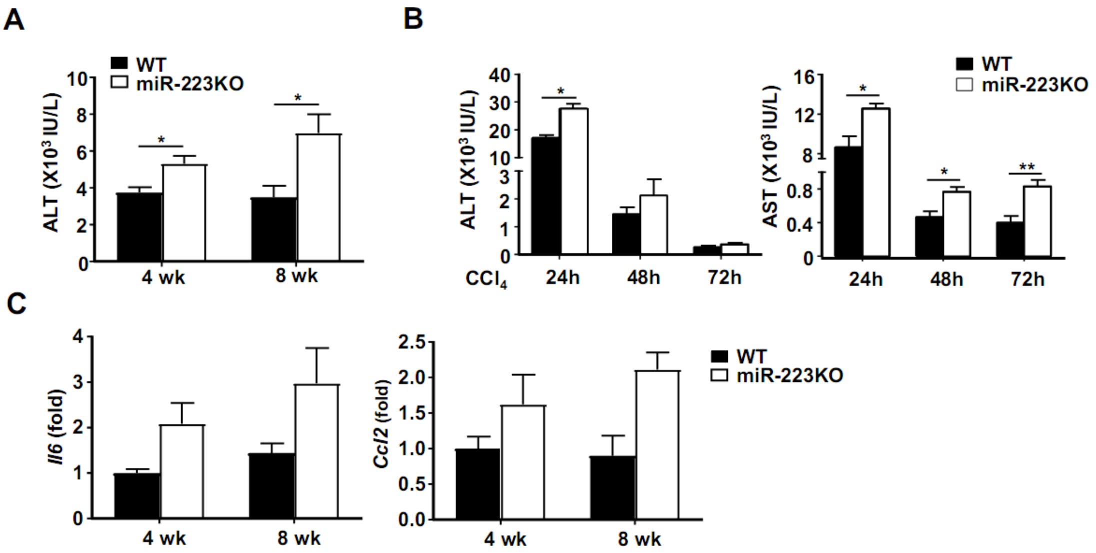

Supplementary Figure S1. MiR-223KO mice are more susceptible to CCl_4-induced liver injury. WT and miR-223KO mice were treated with CCl_4 twice a week for 4 weeks or 8 weeks (n=5-8/group) (A,C). (A) The ALT levels were measured. (B) WT and miR-223KO mice were treated with CCl_4 for the indicated time points. Serum ALT and AST levels were measured. (C) RT-qPCR analyses of the expression of *Il6* and *Ccl2* in the liver from WT and miR-223KO mice. Values represent means ± SEM. $^{*}P< 0.05$, $^{**}P< 0.01$.

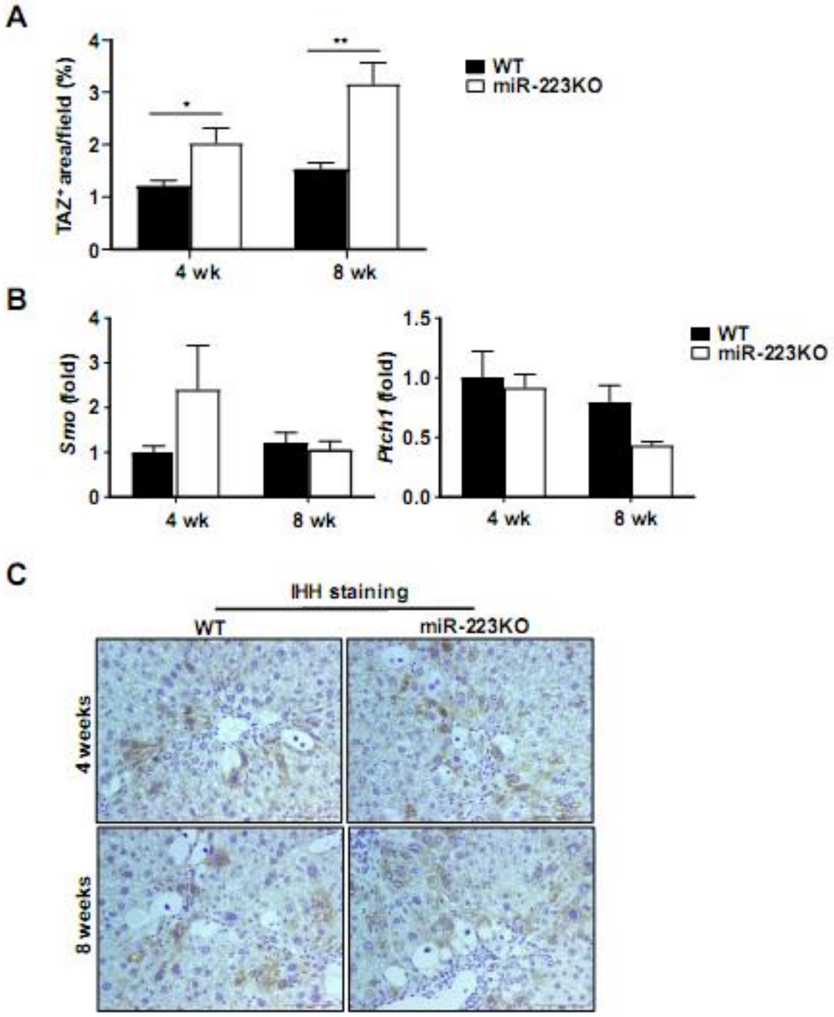

Supplementary Figure S2. MiR-223KO mice show higher levels of IHH expression after chronic CCl_4 injection. WT and miR-223KO mice were treated with CCl_4 twice a week for 4 weeks or 8 weeks (n=5-8/group). (A) Quantification of TAZ^+ area per field. (B) RT-qPCR analyses of the expression profiles of the genes related to Hedgehog signaling pathway in the liver from WT and miR-223KO mice. (C) Representative images of IHH staining are shown. Values represent means ± SEM. $^*P < 0.05$, $^{**}P < 0.01$.

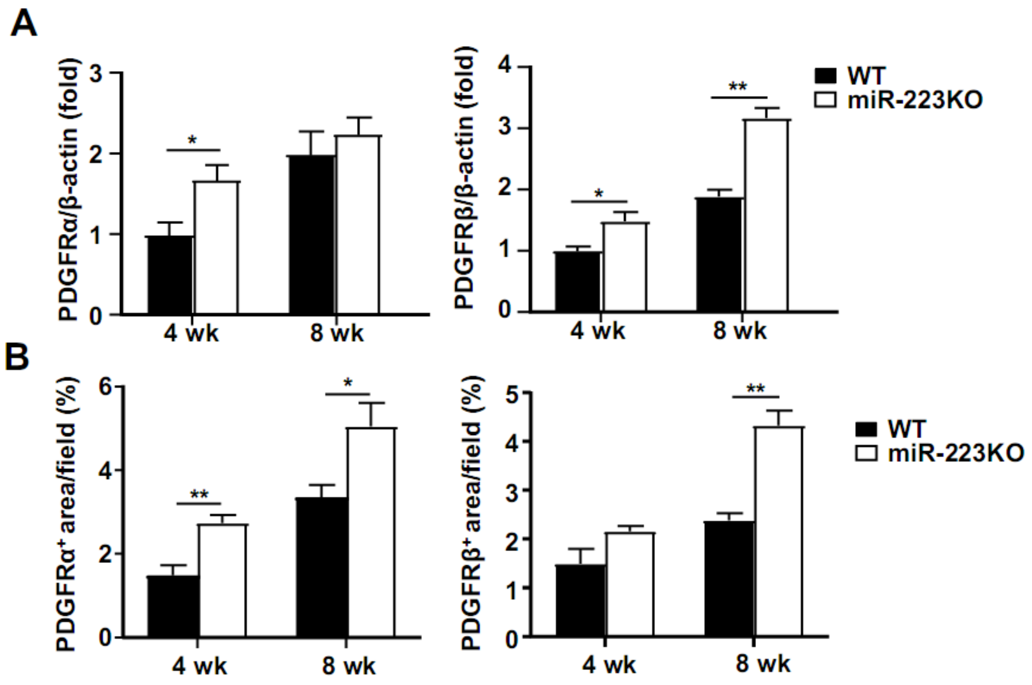

Supplementary Figure S3. **Expression quantification of several proteins in western blot and immunohistochemistry staining as shown in Fig. 4B and 4C**. Values represent means ± SEM. **P*< 0.05, ***P*< 0.01 as compared to WT group.

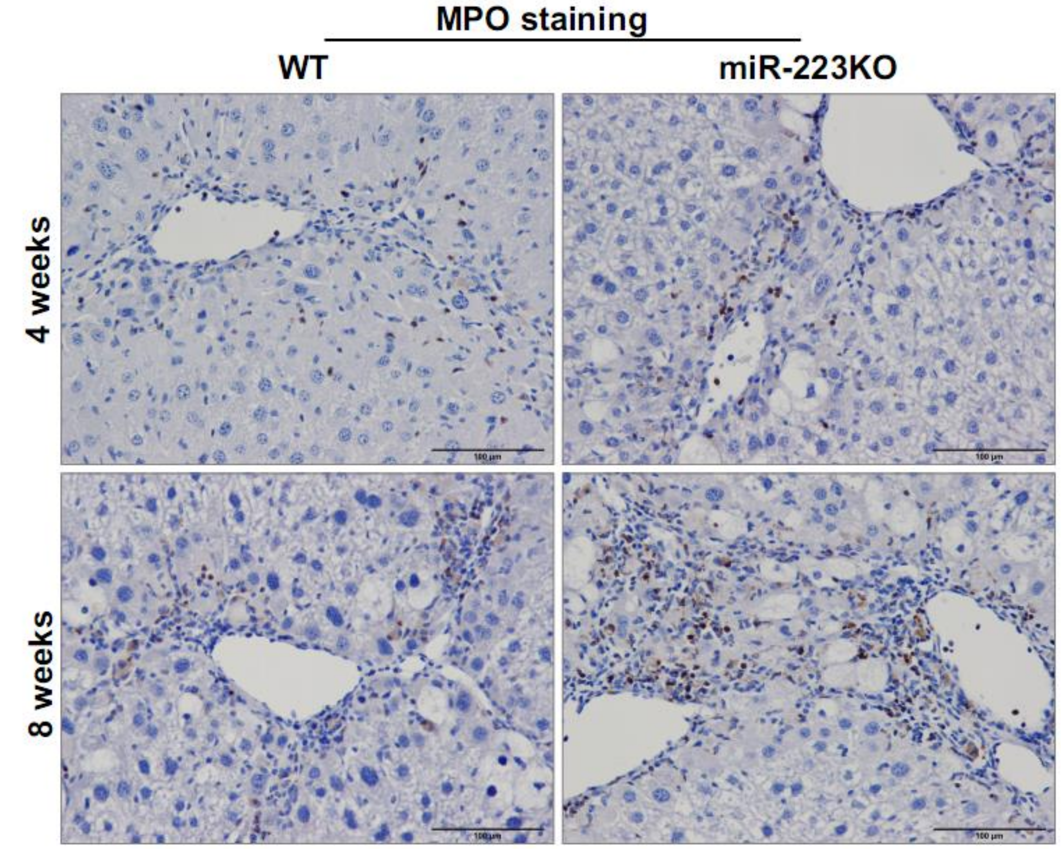

Supplementary Figure S4. MiR-223KO mice have more liver neutrophil infiltration after chronic CCl_4 injection. WT and miR-223KO mice were treated with CCl_4 twice a week for 4 weeks or 8 weeks. Representative images of MPO staining are shown.

Supporting Table 1. Primer sequences for mouse and human genes used in reverse transcription-qPCR.

Mouse Genes	Forward primer (5′–3′)	Reverse primer (5′–3′)
Il6	TCCATCCAGTTGCCTTCTTG	TTCCACGATTTCCCAGAGAAC
Ccl2	CCAGCCTACTCATTGGGAT	GGGCCTGCTGTTCACAGTT
Ccl3	TGAGAGTCTTGGAGGCAGCGA	TGTGGCTACTTGGCAGCAAACA
Ccl4	AACACCATGAAGCTCTGCGT	AGAAACAGCAGGAAGTGGGA
Acta2	TCCTGACGCTGAAGTATCCGATA	GGTGCCAGATCTTTTCCATGTC
Col1a1	TAGGCCATTGTGTATGCAGC	ACATGTTCAGCTTTGTGGACC
Col1a2	GGTGAGCCTGGTCAAACGG	ACTGTGTCCTTTCACGCCTTT
Col3a1	TAGGACTGACCAAGGTGGCT	GGAACCTGGTTTCTTCTCACC
Col4a2	GACCGAGTGCGGTTCAAAG	CGCAGGGCACATCCAACTT
Mmp-13	CTTTGGCTTAGAGGTGACTGG	AGGCACTCCACATCTTGGTTT
Vim	TCCACTTTCCGTTCAAGGTC	AGAGAGAGGAAGCCGAAAGC
Ccnd1	GCGTACCCTGACACCAATCTC	CTCCTCTTCGCACTTCTGCTC
Ccne1	TCCACGCATGCTGAATTATC	TTGCAAGACCCAGATGAAGA
Tgfb1	GACCCTGCCCCTATATTTGGA	CCGGGTTGTGTTGGTTGTAGA
18s	AACTTTCGATGGTAGTCGCCGT	TCCTTGGATGTGGTAGCCGTTT
Gli2	GTTCCAAGGCCTACTCTCGCCTG	CTTGAGCAGTGGAGCACGGACAT
Gli3	CACAGCTCTACGGCGACTG	CTGCATAGTGATTGCGTTTCTTC
Pdgfra	TCCATGCTAGACTCAGAAGTCA	TCCCGGTGGACACAATTTTTC
Pdgfrb	AGGGGGCGTGATGACTAGG	TTCCAGGAGTGATACCAGCTT
Opn	TGGCTATAGGATCTGGGTGC	ATTTGCTTTTGCCTGTTTGG

Human genes	Forward primer (5′–3′)	Reverse primer (5′–3′)
ACTA2	GTGACGAAGCACAGAGCAAA	CTTTTCCATGTCGTCCCAGT

COL1A1	CAGATCACGTCATCGCACAA	TGTGAGGCCACGCATGAG
COL3A1	AGGACTGACCAAGATGGGAA	AGGGGAGCTGGCTACTTCTC
COL4A1	CCTTTTGTCCCTTCACTCCA	CTCCACGAGGAGCACAGC
18S	GGCCCTGTAATTGGAATGAGTC	CCAAGATCCAACTACGAGCTT
TGFB1	CTTCCAGCCGAGGTCCTT	CCCTGGACACCAACTATTGC
VIM	ATTCCACTTTGCGTTCAAGG	CTTCAGAGAGAGGAAGCCGA
CCNE	TCTTTGTCAGGTGTGGGGA	GAAATGGCCAAAATCGACAG
